# BIMSSA: enhancing cancer prediction with salp swarm optimization and ensemble machine learning approaches

**DOI:** 10.3389/fgene.2024.1491602

**Published:** 2025-01-06

**Authors:** Pinakshi Panda, Sukant Kishoro Bisoy, Amrutanshu Panigrahi, Abhilash Pati, Bibhuprasad Sahu, Zheshan Guo, Haipeng Liu, Prince Jain

**Affiliations:** ^1^ Department of Computer Science and Engineering, C. V. Raman Global University, Bhubaneswar, Odisha, India; ^2^ Department of Computer Science and Engineering, Siksha ‘O’ Anusandhan (Deemed to be University), Bhubaneswar, Odisha, India; ^3^ Department of Information Technology, Vardhaman College of Engineering (Autonomous), Hyderabad, Telangana, India; ^4^ Key Laboratory of Biomedical Engineering of Hainan Province, School of Biomedical Engineering, Hainan University, Sanya, China; ^5^ Centre for Intelligent Healthcare, Coventry University, Coventry, United Kingdom; ^6^ Department of Mechatronics Engineering, Parul Institute of Technology, Parul University, Vadodara, Gujarat, India

**Keywords:** cancer prediction, microarray data, feature selection, swarm intelligence, ensemble learning

## Abstract

**Background:**

Cancer rates are rising rapidly, causing global mortality. According to the World Health Organization (WHO), 9.9 million people died from cancer in 2020. Machine learning (ML) helps identify cancer early, reducing deaths. An ML-based cancer diagnostic model can use the patient’s genetic information, such as microarray data. Microarray data are high dimensional, which can degrade the performance of the ML-based models. For this, feature selection becomes essential.

**Methods:**

Swarm Optimization Algorithm (SSA), Improved Maximum Relevance and Minimum Redundancy (IMRMR), and Boruta form the basis of this work’s ML-based model BIMSSA. The BIMSSA model implements a pipelined feature selection method to effectively handle high-dimensional microarray data. Initially, Boruta and IMRMR were applied to extract relevant gene expression aspects. Then, SSA was implemented to optimize feature size. To optimize feature space, five separate machine learning classifiers, Support Vector Machine (SVM), Random Forest (RF), Extreme Learning Machine (ELM), AdaBoost, and XGBoost, were applied as the base learners. Then, majority voting was used to build an ensemble of the top three algorithms. The ensemble ML-based model BIMSSA was evaluated using microarray data from four different cancer types: Adult acute lymphoblastic leukemia and Acute myelogenous leukemia (ALL-AML), Lymphoma, Mixed-lineage leukemia (MLL), and Small round blue cell tumors (SRBCT).

**Results:**

In terms of accuracy, the proposed BIMSSA (Boruta + IMRMR + SSA) achieved 96.7% for ALL-AML, 96.2% for Lymphoma, 95.1% for MLL, and 97.1% for the SRBCT cancer datasets, according to the empirical evaluations.

**Conclusion:**

The results show that the proposed approach can accurately predict different forms of cancer, which is useful for both physicians and researchers.

## 1 Introduction

Cancer has been one of the main causes of mortality across the globe for many decades, making it a significant public health issue on a global scale. Cancer is an umbrella term for illnesses caused by the unchecked proliferation and metastasis of aberrant cells. It significantly affects public health and affects individuals of all ages and walks of life. Cancer mortality rates change both annually and geographically. WHO and the International Agency for Research on Cancer (IARC) frequently release statistics on cancer fatalities. It was predicted that there were around 9.9 million deaths from cancer globally in 2020 (Panigrahi et al., 2023). Deaths from cancer are not distributed in the same way all across the world. Developed nations often have higher incidence and survival rates because of better healthcare, earlier diagnosis, and more effective treatments. However, cancer may be a significant problem in locations with inadequate healthcare infrastructure (Venkatesan et al., 2022). Screening for cancer at an early stage is another way to boost treatment success. Many forms of cancer now have better survival rates because of advances in cancer research and therapy (Khalsan et al., 2022).

Infants with ALL rearranged to MLL have even worse prognoses, with survival rates of 40%–50%; the overall death rate for ALL is 20%–25% in children and up to 50% in adults. The death rate for lymphomas varies greatly depending on subtype. For example, aggressive non-Hodgkin lymphomas, such as diffuse large B-cell lymphoma, have a mortality rate of 30%–40%, whereas Hodgkin lymphomas have a rate below 10%. In children, the survival rate for leukemias with MLL rearrangements is around 50%, but in adults, it is lower, especially in cases of acute myeloid leukemia (AML) (Lewis et al., 2020). The 5-year survival rate for SRBCTs drops to 15%–30% in metastatic instances from over 70% in localized illness (de Leval and Jaffe, 2020).

Through enhancing early detection and diagnosis, treatment, and patient outcomes, machine learning has the potential to contribute significantly to reducing cancer mortality rates (Bolón-Canedo et al., 2014). Medical imaging like mammograms and CT scans may be analyzed using machine learning algorithms for the early detection of cancer. Earlier diagnosis is associated with better treatment results (Ma et al., 2020). It may ensure that patients get the best possible treatment promptly by lowering the percentage of erroneous diagnostic results. Machine learning may predict treatment outcomes by analyzing a patient’s medical record, genetic data, and tumor features (Alghunaim and Al-Baity, 2019). By aiding in medication research and development, machine learning may help produce more effective and tailored medicines for cancer.

Several forms of medical data have been analyzed and interpreted using machine learning (ML) for cancer detection. Medical imaging data and genomic data account for the vast majority of ML-based cancer research. Mammograms, X-rays, CT scans, and MRI scans are all examples of medical imaging data (Almugren and Alshamlan, 2019). DNA sequencing data and gene expression profiling or microarray data are further examples of “genomic data.” Microarray data, or genetic information, is crucial for cancer diagnosis because it reveals important information about the disease itself and its genetic disorders. This knowledge facilitates personalized therapy, boosts treatment efficacy, and facilitates educated decision-making in cancer care and prevention (López-García et al., 2020). When processing microarray data, ML encounters several challenges. The most difficult aspects of the microarray data for ML to handle are the high dimensionality, small sample size, and class imbalance. The high dimensionality of microarray data results from the fact that thousands of characteristics (genes or probes) are often assessed for each sample or patient. Owing to the “curse of dimensionality,” conventional ML methods may become ineffective in required resources (both computational and cognitive) (Shukla et al., 2020a). There are often fewer samples available than there are characteristics to analyze. Creating reliable and generalizable models might be difficult when working with a small sample size.

The best possible solution is to overcome the above-stated challenges, employing strategies such as dimensionality reduction, feature selection, and optimization algorithms to effectively select genes for cancer diagnosis (Shukla et al., 2019). Deploying a single feature selection algorithm may reduce the number of features to some extent. However, if the feature space is larger, employing a single method to select the appropriate number of features may not be sufficient. Hence, the current work aims to deal with the high dimensionality issues of microarray data with the help of the pipelined featured selection algorithms followed by a nature-inspired optimization algorithm to reduce the feature space to the extent upon which the machine learning models can be used to develop a more effective ML model. The reported research includes the Boruta and IMRMR feature selection algorithm in a pipelined manner. The motivation behind using the Boruta Feature selection algorithm is its ability to identify all relevant genes while considering the complex feature interaction with the target feature using Random Forest (RF). In addition, it also deals with the challenges, including the overfitting and interaction of the features with the target variable. Improved Maximum Relevance and Minimum Redundancy (IMRMR) IMRMR improves traditional mRMR by selecting feature subsets highly related to the target class and mainly uncorrelated. This makes features more informative and efficient. This balance is a significant benefit of the approach in complicated datasets where inter-feature relationships may not be evident and impair each feature’s prediction ability. The Salp Swarm Optimization Algorithm (SSA) effectively lowers the dimensionality of datasets and enhances model performance by identifying the most influential features that contribute to the model’s predictive capability. Its innate simplicity and resilience make it highly suitable for managing intricate, high-dimensional data where conventional feature selection approaches may encounter difficulties. It utilizes the swarming characteristics of the salp in an ocean, which balances the exploration and exploitation with efficacy, which helps the SSA to converge on a globally optimal solution while avoiding the local optima. In the current work, BIMSSA, the Boruta is initially applied to eliminate the irrelevant features from the dataset, thus reducing the feature space. It creates the shadow features by shuffling the original features randomly. Using the base classifier RF, the importance score is calculated for original and shadowed features. The highest score of the shadowed feature set is termed the threshold value. The original features with a score greater than the threshold value are considered relevant. Then, the IMRMR is used to select the most relevant features from the feature selection by calculating the IMRMR score. Finally, to the selected features, the SSA is applied to select an optimal number through multiple iterations of features by having a stopping criterion, such as maximum iteration for current work. The algorithms mentioned above are dedicated to selecting the features from the entire feature space. Thus, these algorithms work in a vertical approach.

### 1.1 Objective

This study aims to create an ensemble ML-based model using the Boruta IMRMR technique to select features and the SSA algorithm to optimize those features. The research’s key contribution is summed up as follows.

• To develop an ensemble ML model for efficient Cancer diagnosis.
• To compare the performance of various feature selection approaches, specifically Boruta and IMRMR, in reducing the dimensionality of the microarray data.
• To access the impact of SSA in optimizing the selected features in the context of the Cancer gene expression data.
• To analyze the performance of hybrid models with feature selection methods along with four conventional ML classifiers.
• To analyze the efficacy of the ensemble learning model over the hybrid model.
• Finally, four different cancer microarray data are considered to evaluate the proposed model.

### 1.2 Literature survey

For the current work, 130 reports are initially identified. Several records are excluded from the study with different steps. Twenty duplicate records are removed before the screening process. The remaining 110 numbers of records are considered for the screening phase. In this phase, ten irrelevant records are excluded. From the screening phase, 100 records are processed for the retrieval phase, out of which 7 records are excluded as those records could not be retrieved. Hence, the remaining 93 full-text records are considered. From the considered full texts, 17 records are excluded as sufficient data for analysis are unavailable. In addition, 20 records are excluded as those dealing with diseases other than cancer, and 7 records are found to be irrelevant to the study. Finally, the remaining 49 numbers of articles are considered for the current work. From the considered records, 20 articles are used in the literature survey part, and the remaining 29 numbers of records are considered in the rest of the manuscript. The Figure in Appendix I shows the current work’s Preferred Reporting Items for Systematic Reviews and Meta-Analyses (PRISMA).

Ibrahim et al. (2017) introduced a novel hybrid approach, the Salp Swarm Algorithm in feature selection (SSA-FS), on the real datasets obtained from Iraqi hospitals for breast, bladder, and colon cancers. Hegazy et al. (2018a) introduced a novel method, the chaotic salp swarm algorithm (CSSA), to enhance the SSA on 28 datasets. Hegazy et al. (2018b) introduced a novel method, the improved salp swarm algorithm (ISSA), which is consolidated with the KNN classifier for feature selection on 23 UCI-ML datasets and claimed to achieve enhanced accuracies on Breast Cancer, Lung Cancer, and BeastEW disease datasets respectively. Using a neighborhood entropy-based uncertainty measures model, Sun et al. (Sun et al., 2019) successfully applied machine learning (ML) methods, including k-nearest neighbor (KNN), C4.5, and Support Vector Machine (SVM), to the classification of the colon, diffuse large B-cell lymphoma (DLBCL), leukemia, lung, and small round blue cell tumor (SRBCT). Ghoniem (2020) introduced a novel bio-inspired liver cancer diagnosis model considering a deep learning (DL) approach, i.e., Convolutional Neural Network (CNN) along with SegNet and UNet, and the optimization technique, i.e., Artificial Bee Colony optimization (ABC) on Radiopaedia and LiTS datasets. Shukla et al. (2020b) introduced an adaptive inertia weight teaching-learning model considering machine learning approaches, i.e., Support Vector Machine (SVM), Extreme Learning Machine (ELM), and Naïve Bayes (NB) on Breast Cancer, Colon Cancer, DLBCL, Leukaemia, SRBCT, Lung Cancer.

Meenachi and Ramakrishnan (2020) introduced differential evolution and global optimal feature selection for cancer data classification model considering the machine learning (ML) approach, i.e., Decision Tree (DT) and optimization technique, i.e., Ant colony optimization (ACO) on five datasets, i.e., DLBCL, Breast Cancer, Leukemia, SRBCT, Gisette datasets. Nouri-Moghaddam et al. (2021) introduced a new hybrid solution based on a multi-filter and adaptive chaotic multi-objective forest optimization algorithm (AC-MOFOA) considering Forest optimization algorithm (FOA), Extreme learning machine (ELM), multi-objective optimization (MOO), and five filter methods, i.e., IG, mRMR, RelifF, CFS, and Fisher-score, on nine datasets, i.e., SRBCT, Tumors_9, Leukaemia3, colon_prostate, Lung, GCM, Breast, Rsctc_5, Rsctc_6.

Yan et al. (2021) introduced a novel feature selection model considering a machine learning (ML) approach, i.e., K-nearest neighbor (KNN) and a wrapper feature selection algorithm FS_SSA based on Salp swarm, on five datasets, i.e., ALL-AML-4, Colon Cancer, Lymphoma, MLL, SRBCT datasets. Sarala et al. (Arun Prabha et al., 2021) introduced a decision-based Salp Swarm Optimization (DT-SWO) algorithm considering machine learning (ML) approaches, i.e., Decision Tree (DT), Support Vector Machine (SVM), Naïve Bayes (NB), Kernel Support Vector Machine (KSVM), and optimization technique, i.e., Salp Swarm Optimization (SWO) on four datasets, i.e., DLBCL, Leukemia, Lung Cancer and colon datasets. Alomari et al. (2021) introduced a hybrid filter-wrapper approach considering robust Minimum Redundancy Maximum Relevancy (rMRMR) as a filter approach, Modified Gray Wolf Optimization (MGWO) as a wrapper approach, and ML approaches including Random Forest (RF), Elastic Networks (EN) and Decision Tree (DT) on nine datasets. Balakrishnan et al. (2021) introduced an improved salp swarm algorithm (iSSA) based on the levy flight for feature selection model and Support Vector Machine (SVM) classifier on six datasets, i.e., Oral Squamous Cell Carcinoma (OSCC), Ovarian cancer, Breast Cancer, CNS, Colon Cancer, Leukemia datasets. Hameed et al. (2021) included the binary particle swarm optimization (BPSO), the genetic algorithm (GA), and the cuckoo search algorithm (CS) for selecting the features. ML approaches, including SVM, NB, KNN, and RF, are applied to twelve datasets.

Gene selection for microarray data categorization was developed by Rostami et al. (2022) using a multi-objective graph theoretic-based approach model that considers the idea of community detection with node centrality (CDNC). On six datasets, Aziz (2022) presented a metaheuristics model that was inspired by nature. The model utilized ML approaches such as Support Vector Machine (SVM), Naïve Bayes (NB), Artificial Neural Network (ANN), cuckoo search (CS), genetic algorithm (GA), and artificial bee colony (ABC). Alromema et al. (2023) used logistic regression, Support Vector Machine, K-Nearest Neighbours, Neural Networks, Naive Bayes, Decision Tree, and eXtreme Gradient Boosting to gene expression datasets. On 17 microarray expression datasets, including CNS, Colon, Leukemia_3C, Leukemia_4C, Leukaemia, Hungtington Disease, DLBCL, Lymphoma66 ö4026_3c, Lymphoma, Prostate, SRBCT, Lung Cancer, Breast Cancer, Sarcoma, Mycloma, and Ovarian, Ke et al. (2022) suggested a population initialization method based on ranking criteria (PIRC) using NB, C4.5, genetic algorithm (GA), and ant colony optimization (ACO). In their study on the Wisconsin Breast Cancer Dataset (WBCD), Rustagi et al. (2024) presented a method for breast cancer detection that relies on Salp Swarm and Grey Wolf Optimisation. They took SVM and KNN classifiers into account. Ünalan et al. (2024) highlighted ensemble learning methodologies’ efficacy in classifying breast cancer. It was revealed that performance improved over standalone classifiers. Classifiers AdaBoost, GBM, and RGF gave an impressive accuracy of 99.5%. However, ensembles of this kind surpassed the respective individual algorithms LGBM for accuracy and gave an F1 score of 99.2% alongside an accuracy of 98.9%. Incorporating stratified shuffle split and k-fold cross-validation raises the question of the strict evaluation technique in obtaining credible and clinically relevant classification outputs. Table 1 shows the analytical study of the literature mentioned above. Batool and Byun (2024) showed that ensemble learning applied to the task of breast cancer classification was doing well by enhancing the general predictive accuracy through a set of multiple models. Several studies on the WBCD dataset showed that the ensemble method, for example, voting classifier involving ETC, LightGBM, RC, and LDA models, performed better than individual models. The proposed model succeeded in surpassing all known state-of-the-art classifiers utilized in the detection and diagnosis cases of breast cancer with an average accuracy of 97.6% and an F1 value of 98.1%. Mahesh et al. (2022) proposes an early prediction of breast cancer through a blended ensemble learning approach with SVM, KNN, DT, RF, and LR as base classifiers. The model’s performance is checked using a breast cancer dataset, which has yielded considerable improvements in accuracy at 98.14%. Accurate, recall, precision, and F1-score metrics validate the ensemble model’s effectiveness over individual classifiers.

**TABLE 1:** Analytical Study of existing literature.

Ref	Techniques employed	Datasets employed	Findings (%)
Ibrahim et al. (2017)	SSA, SVM	Breast Cancer	Accuracy: 98.75
		Bladder	Accuracy: 100
		Colon Cancer	Accuracy: 99.75
Hegazy et al. (2018a)	SSA, KNN	Breast Cancer	Accuracy: 97.08
		Lung Cancer	Accuracy: 60
		Breast EW	Accuracy: 97.08
Hegazy et al. (2018b)	ISSA, KNN	Breast Cancer	Accuracy: 95.70
		Lung Cancer	Accuracy: 59.78
		Breast EW	Accuracy: 96.10
		DLBCL	Accuracy: 92.7
		Leukemia	Accuracy: 92.9
		Lung	Accuracy: 98.8
		SRBCT	Accuracy: 93.6
Sun et al. (2019)	KNN, C4.5, SVM	SRBCT	Accuracy: 93.6
Ghoniem (2020)	CNN along with SegNet and UNet, ABC	Radiopaedia datasets	Accuracy: 99.3, F1-Score: 99.0, Specificity: 99.0
Shukla et al. (2020b)	SVM, ELM, and NB	Breast Cancer	Accuracy: 89.59
		Colon Cancer	Accuracy: 98.03
		DLBCL	Accuracy: 99.89
		Leukaemia	Accuracy: 98.99
		Lung Cancer	Accuracy: 98.83
Meenachi and Ramakrishnan (2020)	DT and ACO	DLBCL	Accuracy: 92.65, Specificity: 98.4, Precision: 95.8, Recall: 95.4, F-Measure: 95.1
		Breast Cancer	Accuracy: 71.88, Specificity: 91.1, Precision: 59.3, Recall: 72.1, F-measure: 64.3
		Leukemia	Accuracy: 85.29, Specificity: 92.8, Precision: 89.6, Recall: 85.8, F-measure: 85.6
		SRBCT	Accuracy: 81.58, Specificity: 93.1, Precision: 82.5, Recall: 82.1, F-measure: 81.7
Nouri-Moghaddam et al. (2021)	FOA, ELM, MOO	SRBCT	Accuracy: 90.72
		Tumors_9	Accuracy: 84.41
		Leukaemia3	Accuracy: 97.66
		Colon	Accuracy: 97.89
		Lung	Accuracy: 93.97
		Breast	Accuracy: 86.53
Yan et al. (2021)	KNN, FS_SSA, PSO, and GA	ALL-AML-4	Accuracy: 94.23
		Colon Tumor	Accuracy: 82.09
		Lymphoma	Accuracy: 88.57
		MLL	Accuracy: 86.19
		SRBCT	Accuracy: 76.74
Arun Prabha et al. (2021)	DT, SVM, NB, KSVM, and SWO	DLBCL	Accuracy: 95
		Leukemia	Accuracy: 97
		Lung Cancer	Accuracy: 94
		Colon	Accuracy: 98
Alomari et al. (2021)	rMRMR-MGWO, LASSO, RF, EN, and DT	Colon Tumor	Accuracy: 94.14, Precision: 95.33, Recall: 91.97, F1-Score: 95.46, Mathew’s Co-relation Coefficient (MCC).: 86.39
		CNS	Accuracy: 100, Precision: 100, Recall: 100, F1- Score: 100, MCC: 100
		AII-AML	Accuracy: 100, Precision: 100, Recall: 100, F1- Score: 100, MCC: 100
		Ovarian Cancer	Accuracy: 100, Precision: 100, Recall: 100, F1- Score: 100, MCC: 100
		Lung Cancer	Accuracy: 97.52, Precision: 94.45, Recall: 98.82, F1-score: 95.79, MCC: 92.0
		ALL-AML-3C	Accuracy: 99.86, Precision: 99.82, Recall: 99.94, F1-Score: 99.77, MCC: 97
		AII-AML-4C	Accuracy: 98.84, Precision: 99.11, Recall: 99.62, F1-Score: 98.63, MCC: 93
		MLL	Accuracy: 99.90, Precision: 99.89, Recall: 99.95, F1-Score: 99.9, MCC: 98.0
Balakrishnan et al. (2021)	SSA, Levy Flight, SVM	OSCC	Accuracy: 85.7, F1-score: 85.7, Recall: 90, Precision: 90.0
		Ovarian Cancer	Accuracy: 83.33, F1-score: 84.3, Recall: 89.5, Precision: 88.0
		Breast Cancer	Accuracy: 50, F1-Score: 66.6, Recall: 100, Precision: 50
		CNS	Accuracy: 66.6, F1-Score: 58.8, Recall: 45.4, Precision: 83.3
		Colon Cancer	Accuracy: 86.9, F1-Score: 88.0, Recall: 100, Precision: 78.5
		Leukemia	Accuracy: 85.7, F1-score: 87.5, Recall: 100, Precision: 100
Hameed et al. (2021)	BPSO, GA, CS, KNN, SVM, NB, RF	Brain	Accuracy: 97.62
		Breast	Accuracy: 86.60
		CNS	Accuracy: 80.00
		Colon	Accuracy: 93.55
		Leukemia	Accuracy: 100
		Lung	Accuracy: 97.54
		Ovarian	Accuracy: 100
		Prostate	Accuracy: 96.08
		TCGA	Accuracy: 100
Rostami et al. (2022)	CDNC	Colon	Accuracy: 88.73
		Leukemia	Accuracy: 90.18
		SRBCT	Accuracy: 82.82
		Prostate Tumor	Accuracy: 82.91
		Lung Cancer	Accuracy: 91.76
Aziz (2022)	SVM, NB, and ANN, along with CS, GA, and ABC	Colon Cancer	Accuracy: 93.01
		Acute Leukemia	Accuracy: 93.35
		Prostate Tumor	Accuracy: 89.14
		High-grade Glioma	Accuracy: 90.32
		Lung Cancer II	Accuracy: 87.71
		Leukemia-2	Accuracy: 93.67
Alromema et al. (2023)	SVM, KNN, NN, NB, DT, XGBoost, LR	Gene Expression Dataset	Accuracy: 97.6, F1-Score: 97.4, AUC:0.961
Ke et al. (2022)	NB, C4.5, GA, and ACO	CNS	Accuracy: 85.00
		Colon	Accuracy: 91.90
		Leukemia_3C	Accuracy: 100
		Leukemia_4C	Accuracy: 97.50
		Leukemia	Accuracy: 100
		DLBCL	Accuracy: 100
		Lymphoma66 × 4,026_3c	Accuracy: 100
		Lymphoma	Accuracy: 93.21
		Prostate	Accuracy: 95.00
		Lung Cancer	Accuracy: 100
		Breast Cancer	Accuracy: 97.18
		Sarcoma	Accuracy: 75.36
		Mycloma	Accuracy: 90.20
		Ovarian	Accuracy: 98.80
Rustagi et al. (2024)	SSA, SVM, KNN	WBCD	Accuracy: 99.42
Ünalan et al. (2024)	AdaBoost, GBM, and RGF, LGBM	WBCD	Accuracy: 99.5
Batool and Byun (2024)	Extra Tree Classifier, Ridge, LGBM	WBCD	Accuracy: 97.6, F-1 Score: 98.1
Mahesh et al. (2022)	SVM, KNN, DT, RF, and LR	Breast Cancer	Accuracy:98.14, Precision: 96.18, Recall, 97.23, and F-1 Score: 96.43

Table 1 shows the analytical study of the considered literature in which it has been observed that the reported literature follows a two-stage feature selection process. In addition, the adopted approaches follow a hybrid approach for cancer classification. Applying the two-stage feature selection does not impact the undertaken dataset more, i.e., ALL-AML, Lymphoma, MLL, and SRBCT cancer dataset. Thus, the current research aims to apply a pipelined feature selection method consisting of three different feature selection approaches, starting from Boruta and followed by IMRMR and SSA. However, to make it more effective, Table 2 has been taken from Table 1, which is dedicated to the literature that deals with the dataset considered for the current study, such as ALL-AML, Lymphoma, MLL, and SRBCT.

**TABLE 2:** Literature survey summary for the ALL-AML, Lymphoma, MLL, and SRBCT datasets.

Ref	Techniques employed	Datasets employed	Findings (%)
Sun et al. (2019)	KNN, C4.5, SVM	SRBCT	Accuracy: 93.6
Meenachi and Ramakrishnan (2020)	DT and ACO	SRBCT	Accuracy: 81.58
Nouri-Moghaddam et al. (2021)	FOA, ELM, MOO	SRBCT	Accuracy: 90.72
Yan et al. (2021)	KNN, FS_SSA, PSO, and GA	Lymphoma	Accuracy: 88.57
		MLL	Accuracy: 86.19
		SRBCT	Accuracy: 76.74
Alomari et al. (2021)	rMRMR-MGWO, LASSO, RF, EN, and DT	ALL-AML-3C	Accuracy: 99.86
Rostami et al. (2022)	CDNC	SRBCT	Accuracy: 82.82
Ke et al. (2022)	NB, C4.5, GA, and ACO	Lymphoma	Accuracy: 93.21

### 1.3 Paper structure

The remaining parts of the paper are organized as follows. Section 2 depicts the approach used in developing the suggested model and describes the utilized dataset. The core ideas behind the proposed paradigm are discussed in Section 3. The empirical evaluation of the suggested model is presented in Section 4. In Section 5, we do a critical analysis of the proposed model. The conclusion is presented in Section 6.

## 2 Methodology of BIMSSA

This section details the methods used to create the reported model and the dataset it was built from. The BIMSSA model utilizes a pipelined feature selection technique to efficiently address the dimensionality problem in the microarray data. Initially, Boruta and IMRMR are used with the objective of extracting pertinent gene expression features. In the next step, the SSA method is used to optimize the size of the feature set acquired by Boruta and IMRMR. The Support Vector Machine (SVM), Extreme Learning Machine (ELM), Random Forest (RF), AdaBoost, and XGBoost are the foundation learners that are used in the construction of an ensemble model.

### 2.1 Dataset description

Four publicly available cancer gene expression data, including ALL-AML (D1), Lymphoma (D2), MLL (D3), and SRBCT (D4), are considered for developing the current work (Zhu et al., 2007). Among the above datasets, D1, D2, and D3 have three classes, and D4 has four classes. ALL-AML dataset contains three classes labeled B-Cell, T-Cell, and AML. The Lymphoma cancer dataset includes DLBCL, FL, and CLL classes. Considering the MLL cancer dataset, it contains three classes, including ALL, AML, and MLL. Similarly, the class names for the SRBCT cancer dataset are BL, EWS, NB, and RMS. Table 3 shows the proposed model’s dataset description and the class-wise distribution in each dataset.

**TABLE 3:** Dataset description.

Dataset	Number of samples	Number of features	Number of classes	Class distribution
ALL-AML	72	7,129	3	B-Cell- 38; T-cell- 9; AML- 25
Lymphoma	62	4,026	3	DLBCL- 46; FL- 9; CLL-11
MLL	72	12,582	3	ALL- 24; AML- 28; MLL- 20
SRBCT	83	2,308	4	BL- 29; EWS- 11; NB- 18; RMS- 25

### 2.2 Boruta feature selection

Boruta is a wrapper-based feature selection that aims to improve the efficiency and interpretability of machine learning models. Boruta finds and chooses important features from a dataset by combining a random forest classifier with a significance testing method. Shadow features, essentially randomized replicas of the original features, are first generated by the algorithm. After that, the original and shadow feature sets are pooled and used to train a random forest model. Based on how each feature affects the model’s performance, Boruta ranks them and compares them to their corresponding shadow characteristics to determine their relative relevance. Initially, dataset (D) contains the total number of features. $\left(F\leftarrow \left\{f_{1},f_{2},\ldots f_{N}\right\}\right)$ , where N is the total samples present in that dataset, then the shadow of the actual features is calculated using Equation 1 to form a shadow feature set (Fshadow):

$$f_{i,shadow}=Shuffle\left(f_{i}\right)=\left[f_{iΡ1},f_{iΡ2},f_{iΡ3},..,f_{iΡN}\right]$$ (1)

Where Shuffle is a function that shuffles the values of the feature $f_{i}$ with a random permutation P with indices {1,2,‥,N}. After getting the shadow features, the D is extended to include the shadow features. Hence, a new feature set ( $F_{integrated}$ ) is derived by integrating F with Fshadow, as represented in Equation 2.

$$F_{integrated}\leftarrow \left\{F,F_{\text{shadow}}\right\}\leftarrow \left\{f_{1},f_{2},\ldots f_{N},f_{1,shadow},f_{2,shadow},\ldots f_{N,shadow}\right\}$$ (2)

To the integrated feature set, $F_{integrated}$ the random forest importance technique is applied to calculate each feature’s importance score(I) using Equation 3.

$$I\left(f_{i}\right)=\sum_{t=1}^{T}\frac{\Delta_{f_{i}}^{t}}{{Κ}_{t}}$$ (3)

After obtaining the I for each feature, the threshold value (Ith) is calculated as the maximum score among the shadow features. This can be represented by Equation 4. Equation 5 shows the criteria based on which the Boruta feature selection algorithms identify the important features present in the dataset, thus discarding the others to modify the feature set (F)

$$I_{th}=\mathrm{Max}\left(I_{f_{shadow}}\right),\forall \;f_{shadow}\in F_{shadow}$$ (4)

$$F\leftarrow \left\{\begin{array}{l} I\left(f_{i}\in F\right)>\;I_{th},Keep\;the\;feature\\ I\left(f_{i}\in F\right)\leq I_{th},Discard\;the\;features \end{array}\right.$$ (5)

Once Boruta determines if a feature is relevant, the algorithm iteratively confirms or rejects it until all features have been categorized. A refined collection of characteristics expected to improve model generalization and prediction accuracy is the final output, a subset of the relevant features. Boruta provides a strong method for feature selection in the ML process, making it ideal for dealing with high-dimensional datasets (Maurya et al., 2023).

### 2.3 Improved maximum relevance and minimum redundancy (IMRMR)

Maximum relevance and minimum redundancy (MRMR) is a filter-based feature selection technique used to select a subset of features from a more extensive set of features. The objective is to select the most useful features while minimizing redundancy. This method is often used in machine learning and data analysis for better model performance, reduced overfitting, and more interpretable models. The IMRMR is the modified version of the MRMR feature section algorithm. Like MRMR, it also selects relevant features with low redundancy scores. MRMR adopts the Mutual Information (MI) to select those features that depend on the target feature. In addition to MI, IMRMR aids Pearson correlation to focus on the linear relation between the features. The following shows the workings of MRMR and IMRMR to determine the difference between these two for selecting features (Ding and Peng, 2005; Yan and Jia, 2019; Zhao et al., 2019).

#### 2.3.1 Relevance calculation

• Calculating each feature’s relevance score requires assessing its correlation with the target variable.
• It is generally accepted that characteristics with high relevance scores should be weighed more for classification.
• The Relevance score ( $M_{re}$ ) of a feature is calculated based on Mutual Information (MI) as Equations 6 and 7.

$$MI\left(f_{i},f_{c}\right)=\sum_{f_{i}\in F}P\left(f_{i},f_{c}\right)*\sum \log_{2}\left(\frac{P\left(f_{i},f_{c}\right)}{p\left(f_{i}\right)*\;p\left(f_{c}\right)}\right)$$ (6)

$${\max\;M}_{re}\left(f_{i},f_{c}\right)=\frac{1}{\left|F\right|}\sum_{f_{i}\in F}\left(MI\left(f_{i},f_{c}\right)\right)$$ (7)

F is the total feature set, |F| is the total number of features, $f_{i}$ is known as the selected feature, $f_{c}$ is the target variable of the feature set. $P\left(f_{i},f_{c}\right)$ is the joint probability of the feature $f_{i}\;and\;f_{c}$ . $p\left(f_{i}\right)*\;p\left(f_{c}\right)$ are the marginal probabilities of the features $f_{i},and\;f_{c}$ respectively (Ke et al., 2022).

#### 2.3.2 Redundancy calculation

• Perform a pairwise redundancy analysis between the characteristics. The word “redundancy” refers to the degree to which two characteristics are comparable regarding the information they contain.
• Metrics such as mutual information, correlation, and distance-based metrics are common redundancy measurements.
• It is best to avoid using features in the final decision that are extremely repetitive with one another since they give comparable information. The redundancy score ( $M_{rd}$ ) can be calculated using Equations 8 and 9.

$$MI\left(f_{i},f_{j}\right)=\sum_{f_{i}\in F}P\left(f_{i},f_{j}\right)*\sum \log_{2}\left(\frac{P\left(f_{i},f_{j}\right)}{p\left(f_{i}\right)*\;p\left(f_{j}\right)}\right)$$ (8)

$$\min\;M_{rd}\left(f_{i},f_{j}\right)=\frac{1}{\left|F\right|}\sum_{f_{i},f_{j}\in F}\left(MI\left(f_{i},f_{j}\right)\right)$$ (9)

The MRMR score of the considered feature $f_{i}$ is calculated as Equation 10. The feature with the highest MRMR score is considered for classification (Rustagi et al., 2024).

$$\text{MRMR}\left(i\right)=\mathrm{Max}\left(M_{\text{re}}-M_{\text{rd}}\right)$$ (10)

By determining the significance and duplication of each feature, the mRMR technique quantifies the contribution of features. It does not take into account the combined effect of several characteristics. Mutual information measures are the only basis for relevance and redundancy. To choose the best feature subset, IMRMR employs two metrics—the Pearson correlation coefficient and mutual information—to assess the subsets’ relevance and redundancy with weight factors ranging [0.1,1] with step size 0.1.

Calculate the relevance ( $M_{re}$ ) and redundancy ( $M_{rd}$ ) of each feature ( $f_{i}$ ) in the feature space F using Equations 11, 13, introducing the Pearson Correlation (Equation 12) and weight factor *α* ranging from [0.1, 1] with step size 0.1.

$$M_{re}\left(f_{i},f_{c}\right)=\propto *\left(MI\left(f_{i},f_{c}\right)\right)+\left(1-\propto \right){Pear}_{c}\left(f_{i},f_{c}\right)$$ (11)

$${Pear}_{c}\left(f_{i},f_{c}\right)=\frac{\sum \left(f_{i}-\bar{f_{i}}\right)\left(\left(f_{c}-\bar{f_{c}}\right)\right)}{\sqrt{\sum \left(f_{i}-\bar{f_{i}}\right)\sum \left(f_{c}-\bar{f_{c}}\right)}}$$ (12)

$$M_{rd}\left(f_{i},f_{j}\right)=\frac{1}{F-1}\sum_{f_{i}\in F-1}\left(\propto *\left(MI\left(f_{i},f_{j}\right)\right)+\left(1-\propto \right){Pear}_{c}\left(f_{i},f_{j}\right)\right)$$ (13)

The modified IMRMR(i) can be calculated using Equation 14.

$$\text{IMRMR}\left(i\right)=\mathrm{Max}\left(M\text{re}-M\text{rd}\right)$$ (14)

### 2.4 Salp swarm optimization algorithm (SSA)

After applying the IMRMR feature selection, the relevant features remain in the dataset. However, applying the IMRMR does not ensure that the number of selected features has a role in the diagnosis process. Some features will be relevant, but removing them does not impact the diagnosis model. The Salp Swarm Optimization Algorithm (SSA), a wrapper-based feature selection algorithm, is implemented to ensure that the optimal set of relevant features is in the selected feature set, through which the processing time of the developed model can be decreased.

The SSA has been implemented to solve optimization challenges. It mimics the group dynamics of salps, sea animals similar to jellyfish. When applied to an optimization issue, SSA seeks to identify the best possible outcome (Mirjalili et al., 2017; Castelli et al., 2022). There are more than 1.2 million known marine creature species. Most of these species have similar habits and traits, including ways of communication, speed of movement, and foraging strategies. The salp’s habitats are notoriously tough to reach, yet scientists think this behavior aids the animals in movement and feeding. Inspired by the coordinated movement of salps (gelatinous sea organisms), the SSA uses natural selection to find optimal solutions. Collective behavior in these species serves as a paradigm for the optimization difficulties SSA seeks to solve (Ibrahim et al., 2018; Thawkar, 2021; Sayed et al., 2018).

#### 2.4.1 Mathematical model

A member of the family Salpidae, salps are found in the ocean. Its cylindrical form and end apertures evoke images of jellyfish, which pump water through their gelatinous bodies using internal feeding filters to propel themselves and eat. Some of the aquatic creatures exhibit similar behaviors, such as swarming. In the case of fish, this group is known as a school, whereas in the case of salps, it is referred to as a salp chain.

The SSA starts with the swarm X of n numbers of salps. Equation 15 shows the two-dimensional matrix of the scalp position.

$$\begin{array}{ccc} X_{11} & \ldots & X_{1n}\\ \vdots & \ddots & \vdots \\ X_{m1} & \ldots & X_{mn} \end{array}$$ (15)

The working of the SSA is started by determining the fitness function (F ()) population size, maximum iteration, initial step size ( $\Gamma_{1}$ )and damping factor ( $\Gamma_{2}$ ). For each salp $X_{i}$ the social influence is calculated as Equation 16 (Thawkar, 2021).

$$S\left(X_{i}\right)=\sum_{i,j}\frac{F\left(X_{i}\right)}{\left|X_{j}-X_{i}\right|}$$ (16)

$F\left(X_{i}\right)$ is the fitness function of the salp $X_{i}$ . $\left|X_{j}-X_{i}\right|$ is the Euclidian distance between two salps $X_{j}\;and\;X_{i}$ . With the personal best solution (Xb), the personal component P is calculated as Equation 17. The exploration component (E) can be calculated as Equation 18.

$$P\left(X_{i}\right)=\frac{F\left(X_{i}\right)}{\left|X_{b}-X_{i}\right|}$$ (17)

$$\left.E\left(X_{i}\right)=\kappa *R\left(\;\right)\left.*\right|X_{b}-X_{i}\right|$$ (18)

Where κ is a random vector between [0,1], and R () is the method to generate the random number between 0 and 1. $X_{b}$ is the personal best of the selected salp. The position of the salp can be updated by using Equation 19.

$$X_{i}\left(new\right)=X_{i}+\Gamma_{1}*S\left(X_{i}\right)+\Gamma_{2}*P\left(X_{i}\right)+E\left(X_{i}\right)$$ (19)

The fitness of the salp is updated by comparing the fitness of the new position with the previous position. This can be determined by using Equation 20. Personal best is based on the fitness function obtained. The position of salp can be obtained by using Equation 21.

$$F\left(X_{i}\left(new\right)\right)=\left\{\begin{array}{l} F\left(X_{i}\right),if\;F\left(X_{i}\left(new\right)\right)<F\left(X_{i}\right)\\ F\left(X_{i}\left(new\right)\right),else \end{array}\right.$$ (20)

$$X_{b}=\left\{\begin{array}{l} X_{i}\left(new\right),if\;F\left(X_{i}\left(new\right)\right)<F\left(X_{i}\right)\\ X_{i},else \end{array}\right.$$ (21)

The exploitation components of salp aim to improve the current obtained solution to an optimized one. This process ensures that the obtained solution is the best. Exploitation is the process of leader-follower dynamics where the entire salp group is divided into two groups. The first salp is the leader salp, and the others are called the follower salp. The position of the leader salp and follower salp is updated using Equations 22 and 23, respectively. Let x, y be the leader and follower salp belonging to the salp community X.

$$x\left(t+1\right)=x\left(t\right)+c1\left(\left(ub-lb\right)c2+lb\right)$$ (22)

Where lb and ub are the lower and upper bounds of the exploring dimension, x(t) is the current position of the leader salp at current time t, and c1 and c2 are the random numbers between the interval [0,1].

$$y_{i}\left(t+1\right)=\frac{1}{2}\left(y_{i}\left(t\right)+y_{i-1}\left(t\right)\right)$$ (23)

$y_{i}\left(t\right)$ is the current position of a follower salp $y_{i}\in X$ , $y_{i-1}\left(t\right)$ is the current position of the proceeding follower salp.

#### 2.4.2 Working of SSA

**FIGURE 1:**
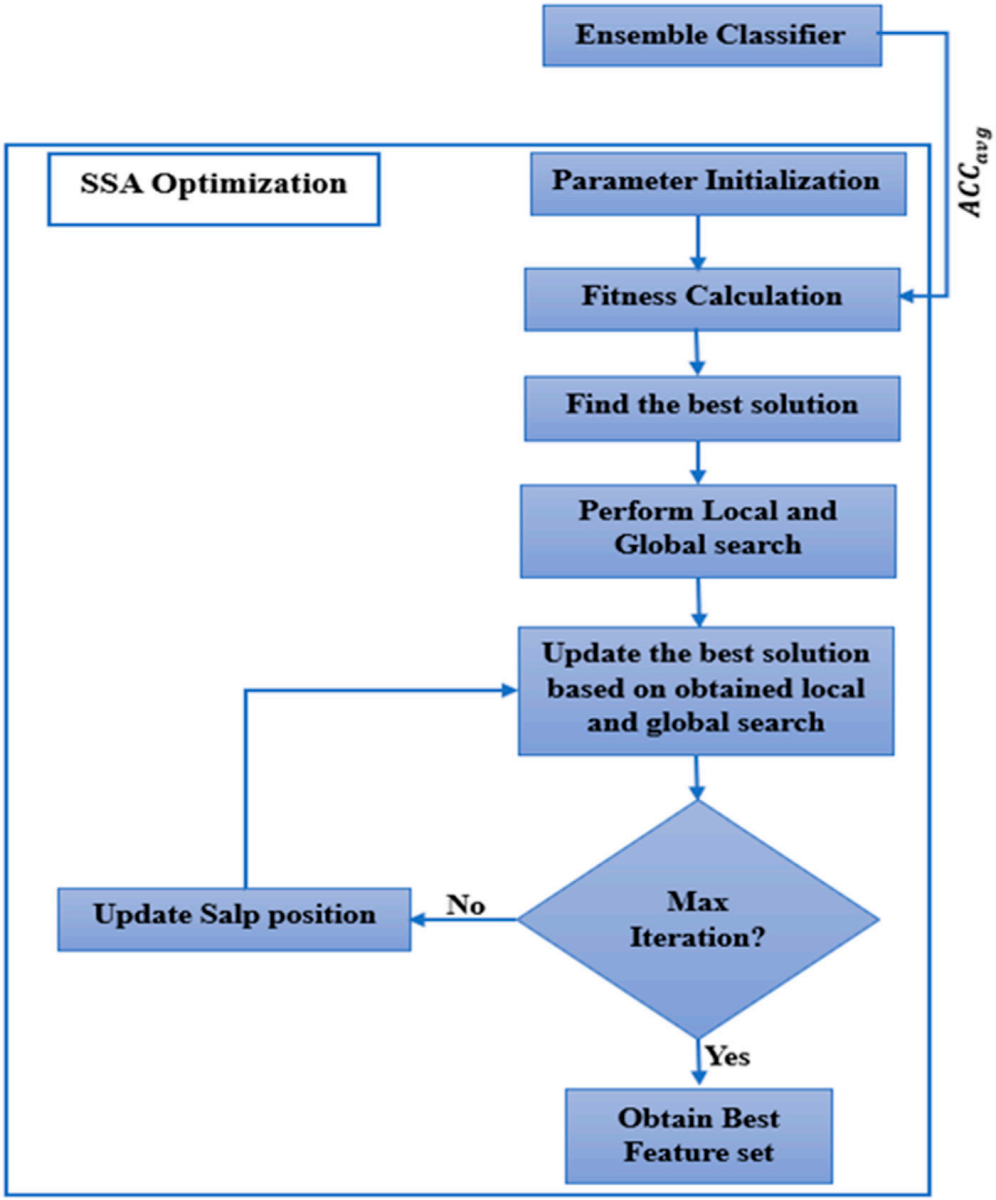
Working of SSA.

The selected features from MRMR are taken as input for the SSA algorithm. The workings of the SSA are described below. Figure 1 shows how the SSA algorithm works.

Step 1: The parameter initialization is the primary of the algorithm. Various parameters have been initiated, including maximum iteration (Max_It), number of salps (num_salps), etc. The parameter initialization is shown in Table 4.
Step 2: To get the best solution, the proper fitness function needs to be calculated. For the current work, average accuracy (Equation 24) determines the fitness function of SSA. For determining the across-validation, the fitness function $F\left(\right)$ can be calculated by using Equation 25.

$${ACC}_{avg}=\frac{1}{k}*\sum_{i}{ACC}_{i}$$ (24)

$$F\left(\right)={ACC}_{avg}$$ (25)

Step 3: The best solution is identified based on the objective function.
Step 4: Update the salp position as per Equation 15.
Step 5: Local and global exploration is performed to find the local and global solution. This algorithm aims to strike a balance between global exploration, which searches the whole solution space, and local exploration, which exploits the neighborhood of specific solutions.
Step 6: Update the best solution.
Step 7: Repeat Step-3 to Step-6 for each feature until the number of iterations is less than Max_It.

**TABLE 4:** Parameter initialization of SSA.

SSA parameter	Value
Max_It	100
num_salps	30
Lb	−10
Ub	10
$\Gamma_{1}$	1
$\Gamma_{2}$	0.8

### 2.5 Voting ensemble classifier

The current work employs ML classifiers such as SVM (Alfian et al., 2022), RF (Alfian et al., 2022), ELM (Ding et al., 2013), AdaBoost (Asselman et al., 2021), and XGBoost (Asselman et al., 2021) to make the initial prediction. Then, the majority voting classifier is used as an ensemble learning approach to make the final prediction. It is a simple and powerful strategy for integrating the results of numerous models into a single prediction. When many models provide varying results and a group choice must be made based on those results, majority voting is an effective tool (Naji et al., 2021; Pati et al., 2023).

## 3 Workflow of BIMSSA

**FIGURE 2:**
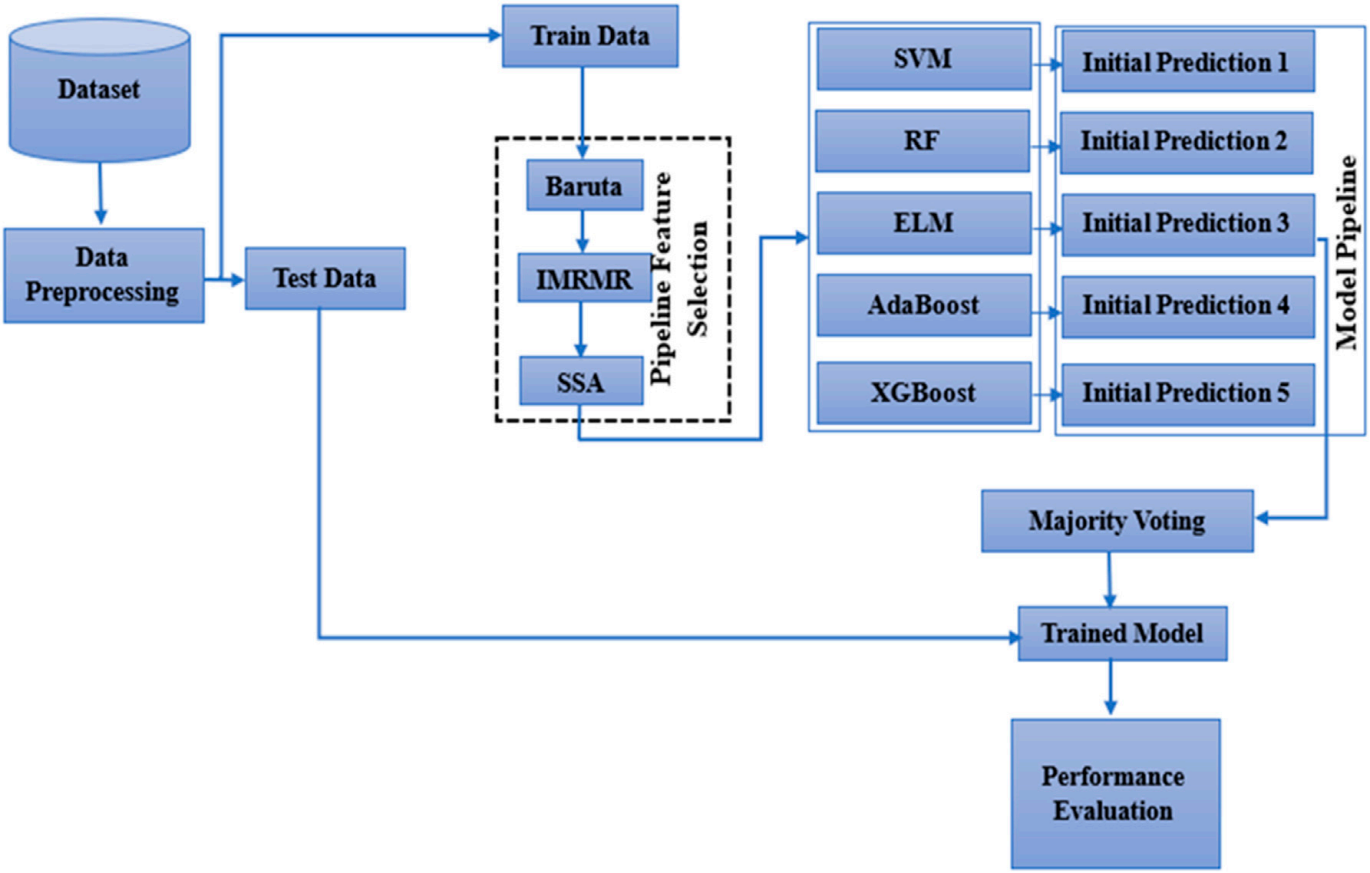
Workflow of the Proposed BIMSSA for feature selection and classification.

Initially, the dataset is considered for normalization to remove the noisy data. The MRMR is then applied to the normalized data to choose the best feature. As a last step, the SSA is used as an optimizer. Algorithm 1 shows the working description of BIMSSA. The functionality of the suggested model is shown in Figure 2. Initially, the dataset is considered for normalization to remove the noisy data. The MRMR is then applied to the normalized data to choose the best feature. As a last step, the SSA is used as an optimizer. Figure 2 and Algorithm 1 show the suggested model’s functionality. The suggested method’s operation is described below.

### 3.1 Step-1: dataset considered for preprocessing

The dataset is first subjected to a preprocessing stage to ensure data quality and consistency. This involves several key steps.

• Data Cleaning: Remove or correct any noisy data, missing values, or inconsistencies within the dataset.
• Normalization: Apply normalization techniques to scale the features to a standard range, typically [0, 1] or [-1, 1]. This helps in improving the performance and convergence of machine learning algorithms.
• Encoding: If applicable, convert categorical variables into numerical values using label encoding.
• Balancing the Dataset: If the dataset is imbalanced, techniques like oversampling, undersampling, or synthetic data generation (e.g., SMOTE) are applied to ensure that the model does not become biased towards the majority class.

### 3.2 Step 2: dataset splitting

The preprocessed dataset is then split into training and testing sets. Two different splitting ratios are considered: 80–20. This helps in evaluating the robustness of the model.

### 3.3 Step 3: Feature selection

To enhance model performance, feature selection algorithms are applied.

• Boruta Algorithm: An all-relevant feature selection method that identifies relevant features by comparing original attributes with shadow attributes.
• Improved Minimum Redundancy Maximum Relevance (MRMR): Select the best features with maximum relevance with the target variable and minimal redundancy among themselves.

### 3.4 Step 4: optimization using salp swarm algorithm (SSA)

The SSA is used to optimize the feature set selected in the previous step.

i. Initiate Population
ii. Parameter Initialization
iii. Fitness Function Calculation
iv. Optimization Process Starts: Iteratively update the positions of salps to find the optimal feature set.
v. Check the Maximum Iteration
vi. If Not Exceeding, Return to iii
vii. If Exceeds, Obtain the Best Feature Set

### 3.5 Step 5: model training

Based on the training data and the optimized feature set, train the following five models.

• Support Vector Machine (SVM)
• Random Forest (RF)
• Extreme Learning Machine (ELM)
• AdaBoost
• XGBoost

### 3.6 Step 6: Classifier Selection

Select the top three classifiers from the trained models based on their highest accuracy during training.

### 3.7 Step 7: Model Evaluation

Test the ensemble classifier using the testing dataset to obtain evaluative parameters such as accuracy, precision, recall, F1-score, and ROC-AUC.

Algorithm 1 Working of Proposed BIMSSA model.

Input: **Dataset D← {D**_**1**_ **, D**_**2**_ **, D**_**3**_ **, D**_**4**_ **}, Feature set F← {f**_**1**_ **, f**_**2**_ **, ……f**_**n**_ **}, Max Iteration, num_salps, Lb, Ub, Γ**_**1**_ **, Γ**_**2**_ **, BL: {SVM (BL**_**1**_ **), RF (BL**_**2**_ **), ELM (BL**_**3**_ **), AdaBoost (BL**_**4**_ **), XGBoost (BL**_**5**_ **),** $D^{\prime }$ **is the feature set selected by Boruta,** $D^{\prime \prime }$ **is the feature selected by IMRMR,** $D^{\prime \prime \prime }$ **is the feature selected by SSA}**
Output: **Performance Measures**
For k = 1 → 4
 Normalize Dk
End for
Split the dataset with an aspect ratio of 85:15 for Train: Test
Invoke Boruta () to D and return the resulting feature subset is $D^{\prime }$

Invoke IMRMR () to $D^{\prime }$ and return the resulting subset $D^{\prime \prime }$

for k = 1 → 4
 for i = 1 → n
  Find $\text{MI}\left(f_{i},f_{c}\right)$ using Equation 1
  
$M_{\text{re}}=\propto *\left(\text{MI}\left(f_{i},f_{c}\right)\right)+\left(1-\propto \right){\text{Pear}}_{c}\left(f_{i},f_{c}\right)$

  for j = i+1 → n
   Find $\text{MI}\left(f_{i},f_{j}\right)$ using Equation 3
   
$M_{\text{rd}}\left(f_{i},f_{j}\right)=\frac{1}{F-1}\sum_{f_{i}\in F-1}\left(\propto *\left(\text{MI}\left(f_{i},f_{j}\right)\right)+\left(1-\propto \right){\text{Pear}}_{c}\left(f_{i},f_{j}\right)\right)$

  End for
  Find $\text{IMRMR}\left(i\right)$ using Equation 9
  
$D_{k}^{\prime }$ ← $f_{i}$

 End for
End For
Invoke SSA () to $D^{\prime \prime }$ and return resulting feature subset $D^{\prime \prime \prime }$

for k = 1 → 4
 for i = 1 → n
   Find Fitness function F ( $X_{i}$ )
   Calculate $S\left(X_{i}\right)$ using Equation 7
   Calculate $P\left(X_{i}\right)$ using Equation 8
   Calculate $E\left(X_{i}\right)$ using Equation 9
   Find $X_{i}\left(\text{new}\right)=X_{i}+\Gamma_{1}*S\left(X_{i}\right)+\Gamma_{2}*P\left(X_{i}\right)+E\left(X_{i}\right)$

   Find $F\left(X_{i}\left(\text{new}\right)\right)$

   Update position salp $X_{i}$

 End for
 Update $D_{k}^{\prime \prime \prime }$ ← $f_{i}$

End for
Apply Majority Voting ensemble technique with the three best BL
Measure the performance of the proposed model
for k = 1 → 4
 for i = 1 → 5
   Apply BLi to $D_{k}^{\prime \prime \prime }$

   Calculate the performance measures
   Find best three BLi
 End for
End for

## 4 Empirical analysis

The suggested model uses Python 3.11 on Ubuntu 20.04 with 32 GB of RAM, an Intel Core i7 CPU, and a 1 TB SSD. The Boruta and IMRMR feature selection techniques extract the important and relevant features from the dataset. After identifying the relevant features, the SSA optimization technique selects a set of relevant features. The implemented Boruta feature selection algorithm selects 2067, 1,043, 3,767, and 643 features for ALL-AML, Lymphoma, MLL, and SRBCT datasets, respectively. While applying the Boruta + IMRMR hybrid feature selection model, it selects 413, 197, 656, and 118 numbers of features for ALL-AML, Lymphoma, MLL, and SRBCT datasets, respectively. Finally, SSA is applied to the feature subset selected by IMRMR, resulting in 57, 29, 194, and 23 numbers of features selected by the hybrid model Boruta + IMRMR + SSA for the final features classification purpose. The empirical analysis has been done in 3 different approaches. Approach 1 shows the performance of all considered classifiers with the IMRMR feature selection algorithm. Approach 2 shows the performance of all considered classifiers with IMRMR and SSA optimizer. Approach 3 shows the performance of the proposed ensemble classifier with the three best classifiers from Approach 2 based on accuracy. The performance in all of the above-mentioned approaches is based on ten different parameters, including Accuracy (ACC), Precision (PRE), Fβ-score (F1-Score (F1-S) and F2 Score (F 2)), Specificity (SPE), Misclassification Rate (MCR), False Negative Rate (FNR), False Positive Rate (FPR), and MCC. Additionally, we employed confidence interval (CI) statistical analysis of the obtained results (Asselman et al., 2021; Naji et al., 2021). The relative importance of precision and recall can be adjusted using the beta parameter of the F-beta score. Precision and recall are of equal value when β = 1 (F1 score). When β is higher than 1 (F 2 score), the model is more sensitive to positive cases since recall is given more weight. As beta decreases (to values of F0.5), the model becomes more concerned with delivering accurate positive predictions. In the proposed model, two values of β have been considered, such as F-1 and F2. The above-said parameters can be calculated by using Equations 26–35. The training set contains 61 features, and the test set contains 11 samples for the ALL-AML dataset. The training and testing set contains 52 and 10 samples for the Lymphoma dataset. The training set contains 61 features, and the test set contains 11 samples for the MLL dataset. Considering the SRBCT dataset, the train set includes 70 and 13 samples in the test set. Equations 26–35 show the calculation of all evaluative parameters.

$$ACC=\left(\frac{\hat{T}+\hat{F}}{\hat{T}+\overset{═}{T}+\hat{F}+\overset{═}{F}}\right)$$ (26)

$$M_{cr}=\left(1-ACC\right)$$ (27)

$$PRE=\frac{\hat{T}}{\hat{T}+\hat{F}}$$ (28)

$$REC=\frac{\hat{T}}{\hat{T}+\overset{═}{F}}$$ (29)

$$SPE=\frac{\overset{═}{T}}{\overset{═}{T}+F}$$ (30)

$$F-1S=\frac{T}{T+\left(\frac{F+\overset{═}{F}}{2}\right)}$$ (31)

$$F\;2=\frac{5*\;\frac{\hat{T}}{\hat{T}+\hat{F}}*\;\frac{\hat{T}}{\hat{T}+\overset{═}{F}}}{4*\;\frac{\hat{T}}{\hat{T}+\hat{F}}+\frac{\hat{T}}{\hat{T}+\overset{═}{F}}}$$ (32)

$$FNR=\frac{\hat{F}}{\hat{T}+\hat{F}}$$ (33)

$$FPR=\frac{\overset{═}{F}}{\overset{═}{T}+\overset{═}{F}}$$ (34)

$$MCC=\frac{\left\{\left(T*\overset{═}{T}\right)-\left(F*\overset{═}{F}\right)\right\}}{\sqrt{\left(\hat{T}+\hat{F}\right)\left(\hat{T}+\overset{═}{F}\right)\left(\overset{═}{T}+\hat{F}\right)\left(\overset{═}{T}+\overset{═}{F}\right)}}$$ (35)

$\hat{T},\text{and }\hat{F}$ are the true and false positives of the confusion matrix. $\overset{═}{T},\text{and }\overset{═}{F}$ are the true and false negatives of the confusion matrix.

### 4.1 Analysis of hybrid models with Boruta, IMRMR and SSA

**FIGURE 3:**
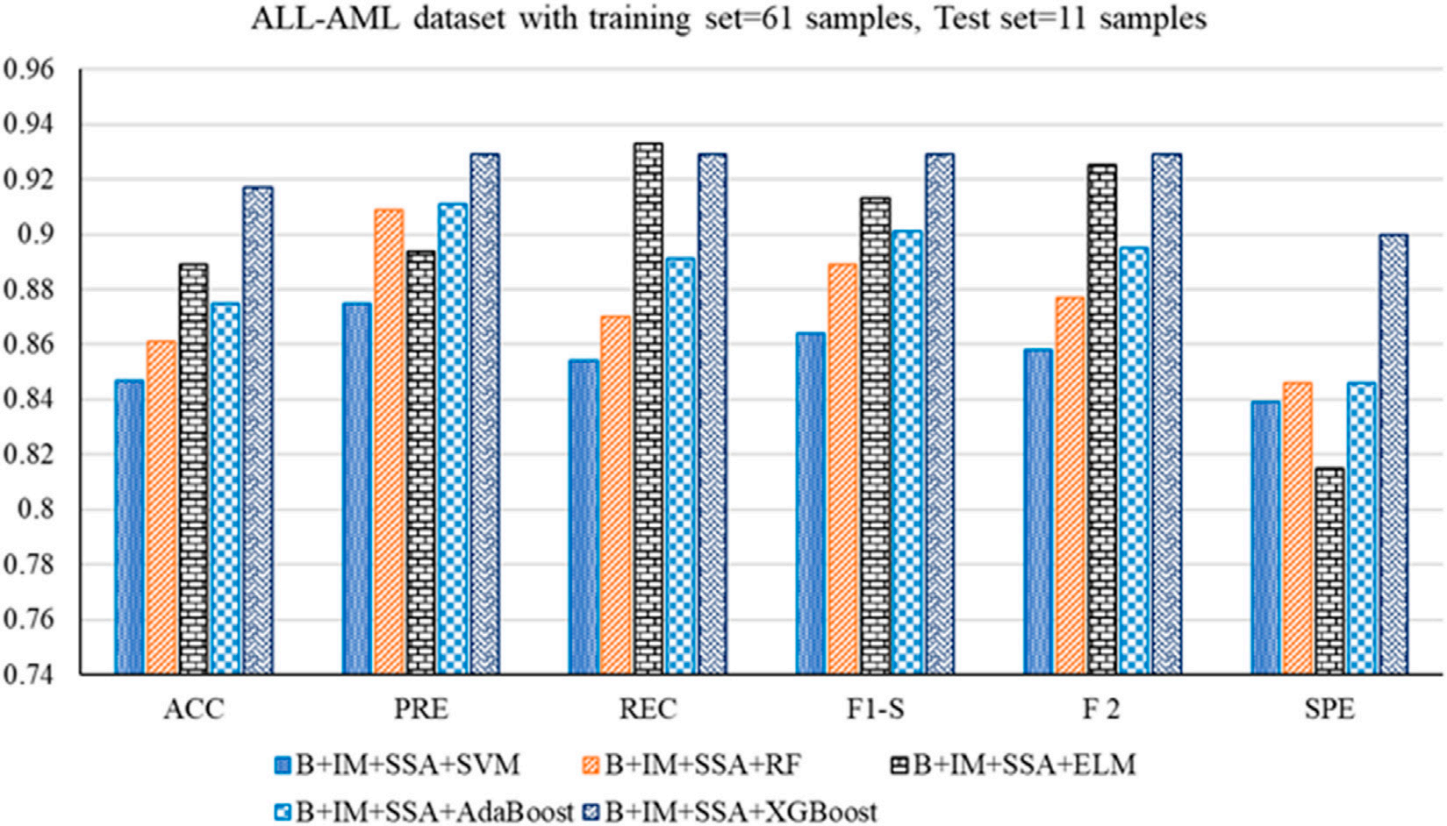
Out-of-sample performance measure of the different classifiers with MRMR and SSA for the ALL-AML dataset.

**FIGURE 4:**
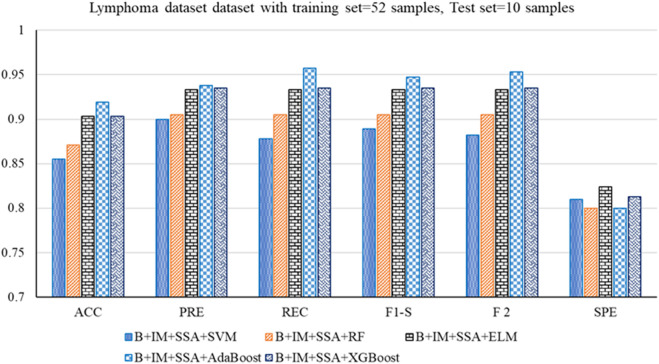
Out-of-sample performance measure of the different classifiers with MRMR and SSA for the Lymphoma dataset.

**FIGURE 5:**
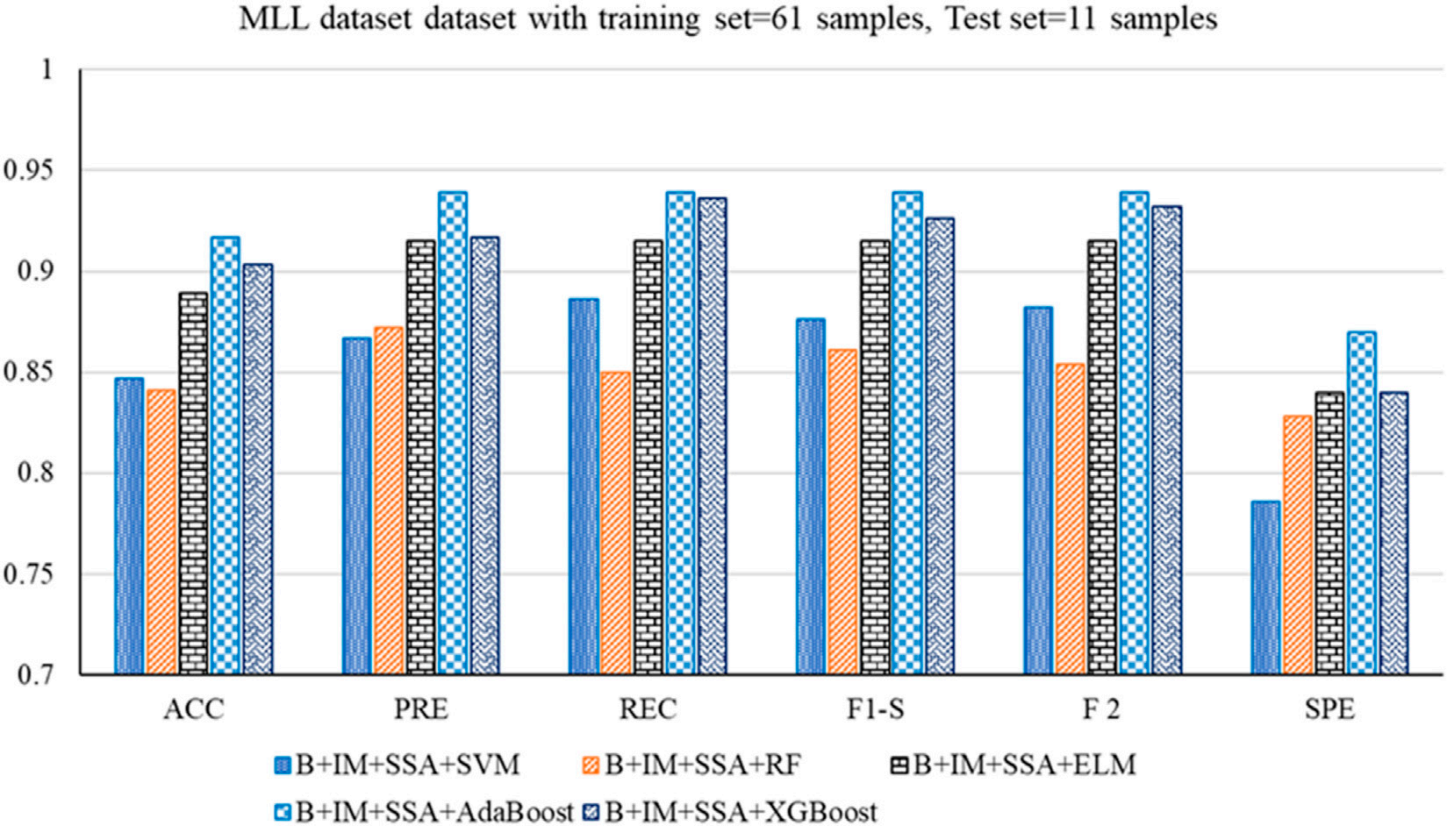
Out-of-sample performance measure of different classifiers with MRMR and SSA for the MLL dataset.

**FIGURE 6:**
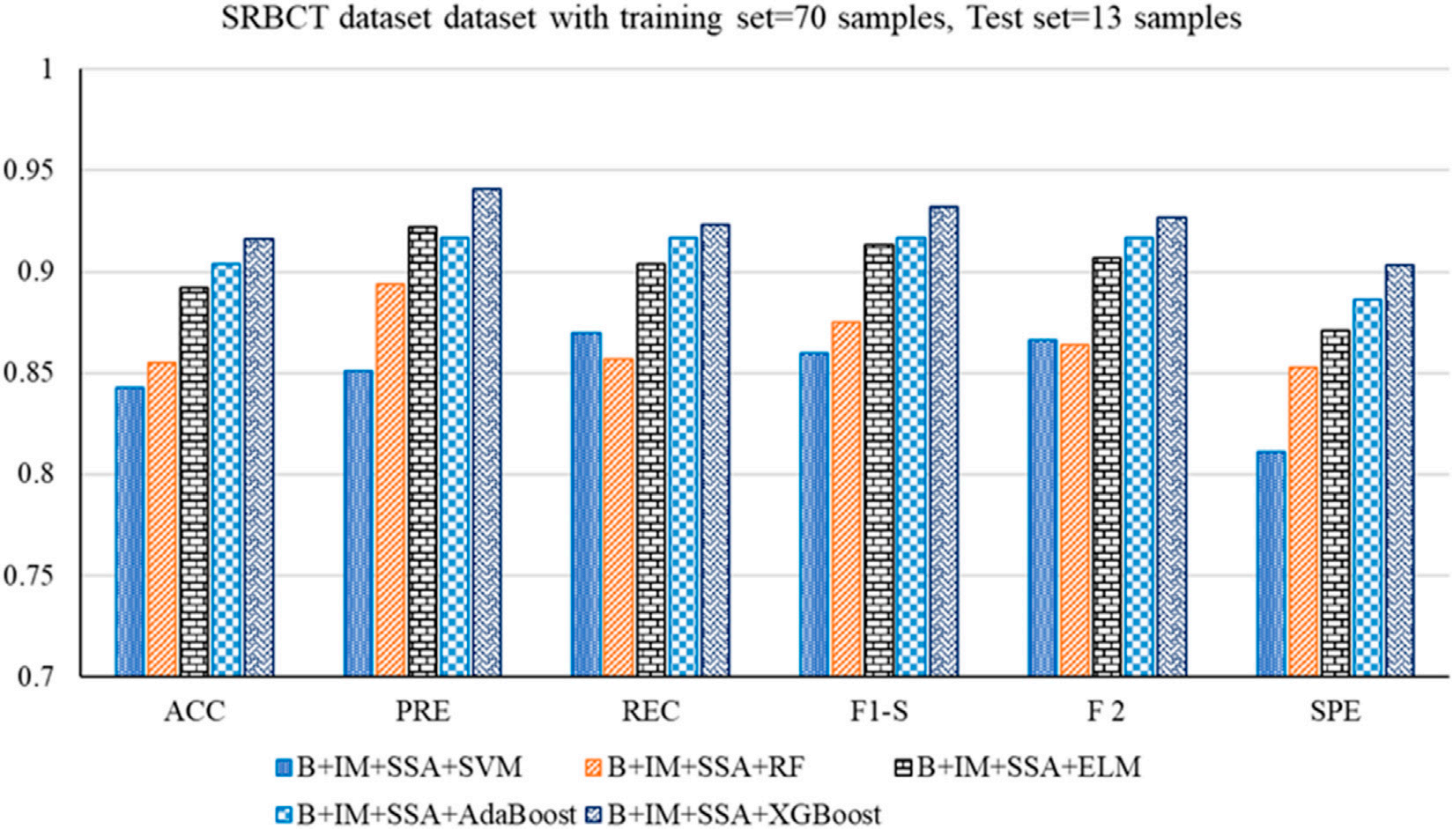
Out-of-sample performance measure of the different classifiers with MRMR and SSA for the SRBCT dataset.

The performance analysis of the mentioned hybrid model, such as Boruta + IMRMR + SSA + SVM, Boruta + IMRMR + SSA + RF, Boruta + IMRMR + SSA + ELM, Boruta + IMRMR + SSA + AdaBoost, Boruta + IMRMR + SSA + XGBoost for different cancer gene expression datasets is summarized as follows. Figures 3–6 show the performance of the hybrid mentioned above models in contrast to different datasets.

• For the ALL-AML dataset, the Boruta (B)+ IMRMR (IM) +XGBoost with SSA shows the highest accuracy at 0.917. The other parameters, such as PRE, REC, F1-S, F 2, and SPE, are 0.929, 0.929, 0.929, and 0.929, respectively. The other parameters, such as FNR, FPR, MCC, and MCR, are 0.071, 0.100, 0.829, and 0.083, respectively.
• For the Lymphoma dataset, B + IM + SSA + AdaBoost shows the best performance with an accuracy of 0.919. The other parameters, such as PRE, REC, F1-S, F 2, and SPE, are 0.938, 0.957, 0.947, 0.953, and 0.800, respectively. The other parameters, such as FNR, FPR, MCC, and MCR, are 0.043, 0.200, 0.776, and 0.081, respectively.
• Similarly, for the MLL dataset, B + IM + AdaBoost with SSA shows the highest performance with an accuracy of 0.917 compared to other hybrid models, as resented. The other parameters, such as PRE, REC, F1-S, F 2, and SPE, are 0.939, 0.939, 0.939, 0.939, and 0.870, respectively. The other parameters, such as FNR, FPR, MCC, and MCR, are 0.061, 0.130, 0.808, and 0.083, respectively.
• With the SRBCT gene expression dataset, the B + IM + SSA + XGBoost performs best with an accuracy of 0.916. The other parameters, such as PRE, REC, F1-S, F 2, and SPE, are 0.941, 0.923, 0.932, 0.927, and 0.903, respectively. The other parameters, such as FNR, FPR, MCC, and MCR, are 0.077, 0.097, 0.821, and 0.084, respectively.

The analysis demonstrates that the B + IM + SSA + AdaBoost model, resulting in an accuracy of 0.919, uses the highest obtained Lymphoma dataset. In order to enhance the accuracy even further, it is possible to include an ensemble machine learning approach. The present study uses the majority voting approach to combine predictions from various models and make a final choice based on the majority of votes. This strategy may improve the accuracy by using the advantages of many models while reducing the limitations of each model. By using majority voting, the ultimate model may amalgamate these advantages, resulting in enhanced overall accuracy and more resilient categorization outcomes. The hybrid models mentioned demonstrate remarkable accuracy across several cancer gene expression datasets, reaching a maximum accuracy of 0.919. A majority voting ensemble classifier may help overcome the problems with the provided hybrid models, namely, their high computational cost and the possibility of overfitting caused by combining several algorithms. By integrating several models’ strengths, this method streamlines decision-making, leads to more accurate forecasts with less hyperparameter tweaking, and reduces the danger of overfitting. To further enhance accuracy, the current work integrates the prediction of the best three classifiers using the majority voting technique. This improves overall precision, minimizes mistakes, and results in more dependable and transferable results in cancer categorization assignments.

### 4.2 Analysis of the proposed model

The performance analysis of the proposed BIMSSA model is shown in Table 5. Table 5 quantifies the ACC, PRE, REC, F-1S, F-2, and SPE measures with Clopper–Pearson confidence interval (CI) (Puza and O’neill, 2006).

**FIGURE 7:**
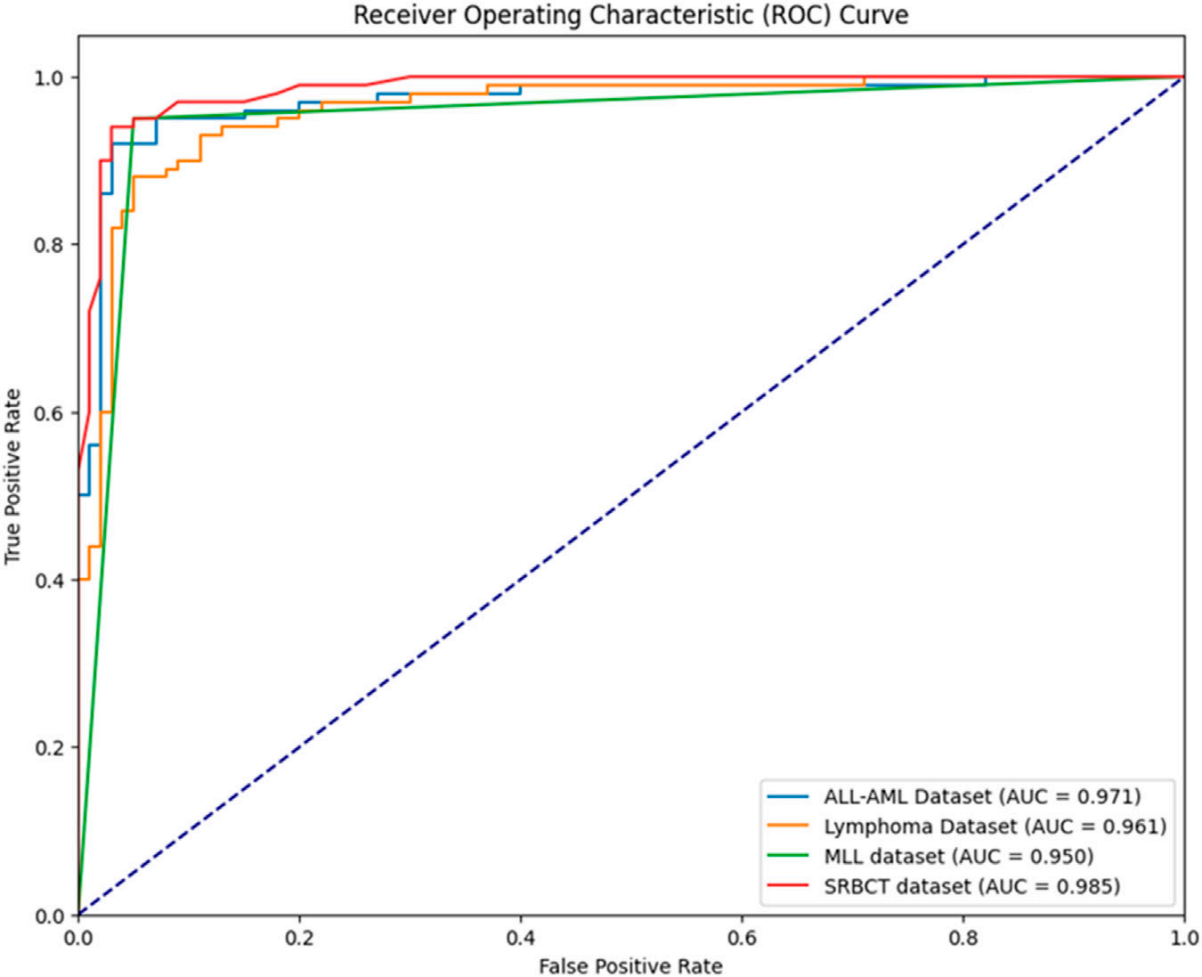
Comparative ROC evaluation for ALL-AML, Lymphoma, MLL, and SRBCT using BIMSSA.

**FIGURE 8:**
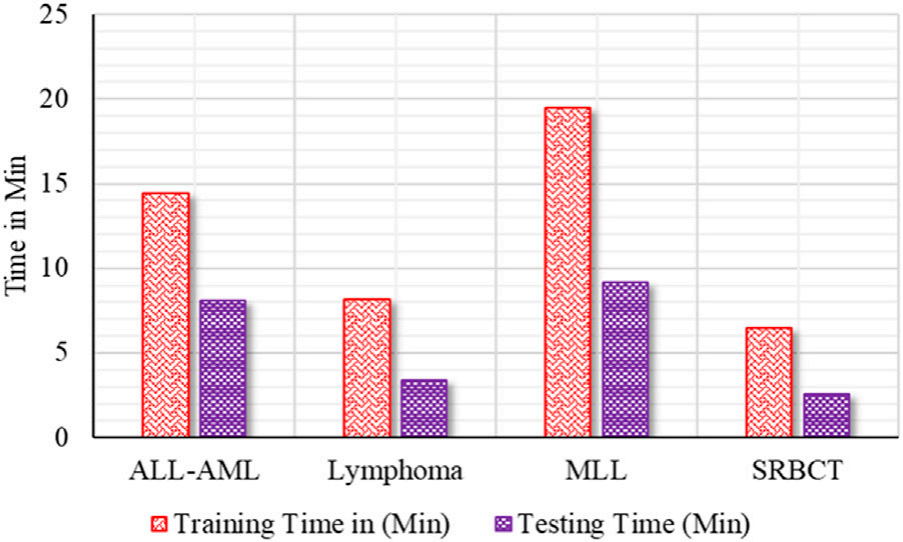
Comparative analysis of Training and Testing Time of BIMSSA over different dataset.

• For the ALL-AML dataset, the proposed BIMSSA model shows the accuracy as 0.967 with a CI of 0.887–0.99. The other parameters, such as PRE, REC, F1-S, F 2, and SPE, are 0.967, 0.974, 0.974, 0.974, 0.974, and 0.955, respectively. The other parameters, such as FNR, FPR, MCC, and MCR, are 0.026, 0.046, 0.929, and 0.033, respectively.
• For the Lymphoma dataset, the proposed BIMSSA shows an accuracy level of 0.962% with a CI of 0.862–0.995. The other parameters, such as PRE, REC, F1-S, F 2, and SPE, are 0.972, 0.972, 0.972, 0.972, and 0.938respectively. The other parameters, such as FNR, FPR, MCC, and MCR, are 0.028, 0.063, 0.910, and 0.039, respectively.
• Similarly, for the MLL dataset, the proposed BIMSSA shows the accuracy level as 0.951 with a CI of 0.887–0.99. The other parameters, such as PRE, REC, F1-S, F 2, and SPE, are 0.949, 0.974, 0.961, 0.969, and 0.913, respectively. The other parameters, such as FNR, FPR, MCC, and MCR, are 0.026, 0.087, 0.895, and 0.049, respectively.
• With SRBCT, the BIMSSA shows a 0.971 accuracy level with a CI of 0.901–0.991. The other parameters, such as PRE, REC, F1-S, and F 2, are 0.978. Specificity (SPE) is 0.958. The other parameters, such as FNR, FPR, MCC, and MCR, are 0.022, 0.042, 0.937, and 029, respectively.
• The model maintains a good level of performance throughout all four datasets (D1, D2, D3, and D4) in regards to accuracy, recall, precision, F1-Score, F2-Score, and specificity. The Clopper-Pearson method’s confidence intervals support the measures’ dependability; narrow intervals show little fluctuation and strong faith in the model’s forecasts. As shown by its low false positive rate (FPR) of 0.042, high Matthews correlation coefficient (MCC) of 0.937, and low Misclassification Rate (MCR) of 0.029, dataset D4 has the best overall performance, especially in accuracy and specificity. These numbers show how well D4 can detect real positives and genuine negatives, which allows it to make strong and trustworthy predictions. D1 additionally demonstrates excellent outcomes; it is a balanced model with a little advantage in predictive accuracy, with a FNR of 0.026, FPR of 0.046, MCC of 0.929, and MCR of 0.033. The great predictive power for D2 is shown in its FNR of 0.028, FPR of 0.063, MCC of 0.910, and MCR of 0.039, which are somewhat lower than D1 and D4, but it still maintains a high level of efficiency. Having a greater FPR of 0.087 affects D3’s MCC of 0.895 and MCR of 0.049, which in turn causes a little drop in specificity. Regardless, the model’s great predictive power and balance between recall and accuracy make it quite useful across all datasets.
• The developed BIMSSA model’s ROC curve is shown in Figure 7 for ALL-AML, Lymphoma, MLL, and SRBCT datasets. The suggested model’s AUC for the ALL-AML dataset is 0.971. The suggested model’s AUC for the Lymphoma dataset is 0.961. The suggested model has AUC values of 0.950 and 0.985 for the MLL and SRBCT datasets.
• Figure 8 shows the training and test time of the developed BIMSSA compared to cancer datasets.

**TABLE 5:** Performance Analysis of Proposed BIMSSA Model with Clopper–Pearson confidence interval (CI).

Dataset	Metric	Predicted value	Clopper–Pearson CI (95%)
D1	ACC	0.967	(0.887, 0.99)
	PRE	0.974	(0.912, 0.996)
	REC	0.974	(0.912, 0.996)
	F1-S	0.974	(0.912, 0.996)
	F-2	0.974	(0.912, 0.996)
	SPE	0.955	(0.863, 0.99)
D2	ACC	0.962	(0.868, 0.995)
	PRE	0.972	(0.897, 0.995)
	REC	0.972	(0.897, 0.995)
	F1-S	0.972	(0.897, 0.995)
	F-2	0.972	(0.897, 0.995)
	SPE	0.938	(0.841, 0.979)
D3	ACC	0.951	(0.887, 0.99)
	PRE	0.949	(0.863, 0.982)
	REC	0.974	(0.912, 0.996)
	F1-S	0.961	(0.887, 0.99)
	F-2	0.969	(0.912, 0.996)
	SPE	0.913	(0.819, 0.963)
D4	ACC	0.971	(0.901, 0.991)
	PRE	0.978	(0.923, 0.997)
	REC	0.978	(0.923, 0.997)
	F1-S	0.978	(0.923, 0.997)
	F-2	0.978	(0.923, 0.997)
	SPE	0.958	(0.88, 0.991)

The proposed BIMSSA does include the Boruta, IMRMR, and SSA as the feature selection algorithm. In addition, the BIMSSA includes SVM, RF, ELM, AdaBoost, and XGBoost as the base classifiers, including majority voting as the ensemble classifier. The model is evaluated over 4 different high dimensional datasets for evaluation. The computational complexity of the proposed BIMSSA model becomes O (T⋅F⋅log(F)) + O(P⋅F⋅I) + O(N^2^⋅F), with N << F. Where, N is the number of sample present in the dataset, F is the number of features, T is the number of trees in the RF classifier, I is the number of iterations, and P is the number of population for SSA.

Although the proposed model is efficient, it still retains several limitations that must be mitigated. First is the increased computational complexity based on the iterative structure of the Boruta algorithm, the added overhead of SSA, and the computation-intensive nature of classifiers used, such as SVM and methods based on trees. Then, hybrid approaches for models increase the complexity of their implementation and call for careful coordination between feature selection and classification phases for optimal performance. The pipeline’s repetitive nature might make it seem to extend training time, making it clumsy for situations requiring deployment and results in a very fast manner.

## 5 Critical analysis

In this section the proposed BIMSSA is compared with some existing literature based on four cancer datasets including ALL-AML, Lymphoma, MLL, and SRBCT. The detailed comparison of BIMSSA with the existing works (Sun et al., 2019; Meenachi and Ramakrishnan, 2020; Nouri-Moghaddam et al., 2021; Yan et al., 2021; Alomari et al., 2021; Rostami et al., 2022; Ke et al., 2022), emphasizing the performance differences across these datasets is given below.

### 5.1 Lymphoma dataset

• For Lymphoma dataset the proposed BIMSSA shows an accuracy of 96.2%, BIMSSA again shows strong performance in classifying Lymphoma, indicating the model’s versatility.
• The work in Yan et al. (2021) reports an accuracy of 88.57% for Lymphoma, which is significantly lower than that of BIMSSA. The proposed model outperforms Yan et al. (2021) by ∼8.61%. This difference may highlight the superiority of the BIMSSA model in handling Lymphoma data, possibly due to better feature selection, model training, and data preprocessing techniques.
• The work in Ke et al. (2022) reports an accuracy of 93.21% for Lymphoma, which is still lower than that of BIMSSA by ∼3.21%. This further confirms BIMSSA’s effectiveness in this context.

### 5.2 MLL dataset

• BIMSSA achieves an accuracy of 95.1%, showcasing its strong performance in this dataset as well.
• The work in Yan et al. (2021) obtains an accuracy of 86.19%, which is lower than that of BIMSSA by a significant margin of ∼10.33%. This difference further emphasizes BIMSSA’s advantage in handling complex datasets.

### 5.3 SRBCT dataset

• BIMSSA with an accuracy of 97.1%, performs exceptionally well on the SRBCT dataset, indicating its robustness and effectiveness across different cancer types.
• The work in Sun et al. (2019) reports a high accuracy of 93.6% for SRBCT, which is noteworthy but still lower than BIMSSA’s accuracy by ∼3.73%.
• In Meenachi and Ramakrishnan (2020) the accuracy is 81.58% which is lower than that of the BIMSSA with a significant margin of ∼19.02%, suggesting that the feature selection, classification and preprocessing techniques used in Meenachi and Ramakrishnan (2020) struggles with this dataset.
• The work in Nouri-Moghaddam et al. (2021) reports an accuracy of 90.72% for SRBCT, which, while remarkable, is still outperformed by BIMSSA by ∼6.92%.
• The work in Yan et al. (2021) reported accuracy here is 76.74%, the lowest among the all compared literature, and BIMSSA outperforms it by ∼26.53%. It is indicating that the model in Yan et al. (2021) may not be as effective for SRBCT.
• In Rostami et al. (2022) the accuracy of 82.82% is reported, which is again significantly low by ∼17.24% when compared to BIMSSA.

### 5.4 ALL-AML dataset

• BIMSSA achieves an accuracy of 96.7%, which demonstrates strong performance and reliability in classifying this cancer type.
• The work in Alomari et al. (2021) reports an exceptionally high accuracy of 99.86%, which outperforms the proposed BIMSSA model by ∼3.27%.

**FIGURE 9:**
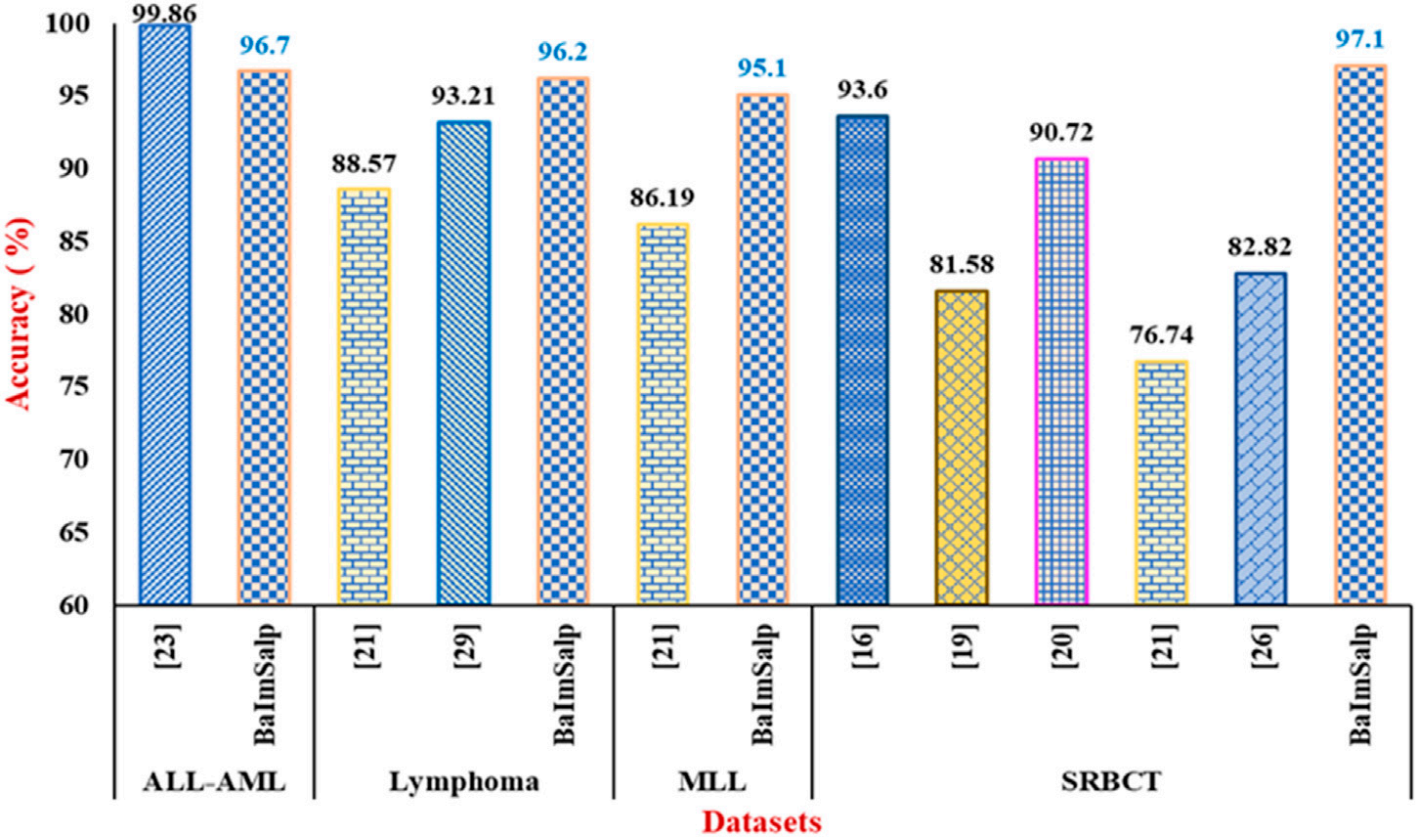
Out-of-sample performance evaluation of BIMSSA with Existing literature.

BIMSSA proves to be a very efficient and dependable model for categorizing cancer, surpassing the accuracy of other models from the existing literature in all tested datasets except ALL-AML. Though the accuracy is low as compared to Alomari et al. (2021) in case of ALL-AML dataset, still it shows an accuracy of 96.7% that can be considered as a noteworthy performance. The model’s constant and superior performance in classifying many forms of cancer, together with its excellent generalization, sets it out as an exceptional model in the field of cancer classification. Therefore, BIMSSA showcases the flexibility and applicability needed for various cancer datasets. Figure 9 shows the comparison of BIMSSA with other existing models in contrast to diverse datasets.

## 6 Conclusion

The primary focus of this study is developing an ensemble machine learning-based model for cancer detection. Gene expression data, also known as microarray data, leaves its unique imprint when used in a cancer detection model. However, there are challenges unique to dealing with microarray data, such as a limited sample size, which diminishes the model’s performance. To deal with this issue, the proposed BIMSSA considers a pipeline feature selection approach with Boruta, IMRMR, and SSA feature selection algorithm to select relevant features. The selected features, such as SVM, RF, ELM, AdaBoost, and XGBoost, are applied to the optimized feature set as the base classifiers. Based on the performance, three classifiers, ELM, AdaBoost, and XGBoost with Boruta, IMRMR, and SSA feature selection, are considered for developing the ensemble model with a majority voting classifier. After selecting three classifiers, we use majority voting to create an ensemble ML-based cancer diagnostic model called BIMSSA. Empirical results from this study using the developed BIMSSA reveal an accuracy of 0.967, 0.962, 0.951, and 0.971 for ALL-AML, Lymphoma, MLL, and SRBCT datasets. The suggested model’s AUC for the ALL-AML dataset is 0.973. The proposed model’s AUC for the Lymphoma dataset is 0.969. The suggested model achieves an AUC of 0.951 for the MLL dataset and 0.979 for the SRBCT dataset. The proposed model does not perform well despite the existing literature regarding the ALL-MLL dataset. However, there are some limitations to the current work. In addition, the current research does not aim to consider the concept of class imbalance.

As a future scope of this manuscript, the feature selection process will be reversed, starting with SSA to explore different combinations of features and identify the promising subset, followed by IMRMR to refine the feature subset further and prioritize the most informative features. Finally, Boruta feature selection is applied to validate the selected feature subset and provide additional insights into the importance of the feature. Testing the model’s performance on more kinds of cancer gene expression datasets is planned for the future of this study. In addition, future plans for this study include using deep learning approaches to increase the model’s capability for working with picture datasets.

## Figures and Tables

**FIGURE A1:**
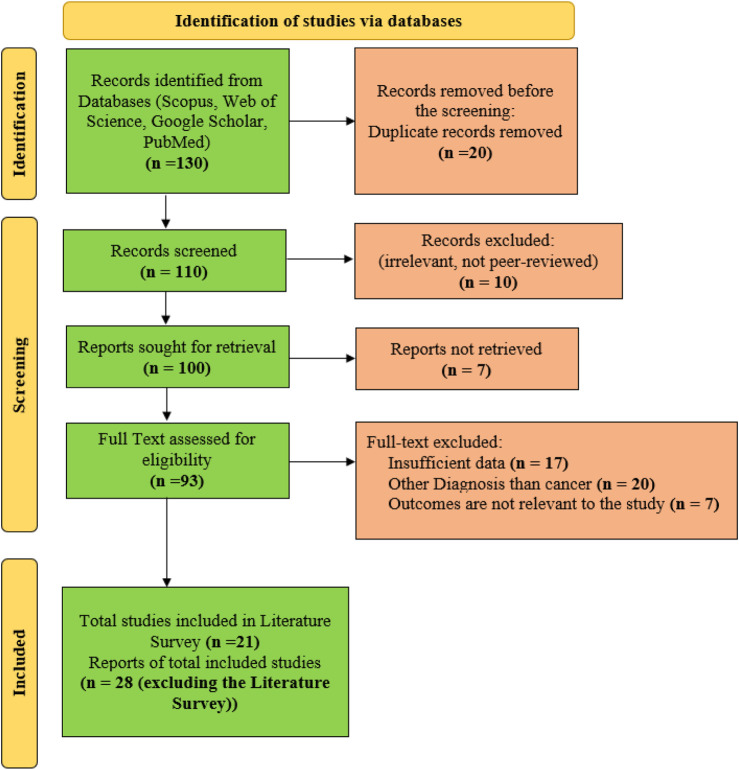
Flow diagram for search strategies and selection of studies for BIMSSA.

## Data Availability

Publicly available datasets were analyzed in this study. This data can be found here: https://csse.szu.edu.cn/staff/zhuzx/Datasets.html.
